# Quality analysis of genomic DNA and authentication of fisheries products based on distinct methods of DNA extraction

**DOI:** 10.1371/journal.pone.0282369

**Published:** 2023-02-28

**Authors:** Ítalo Lutz, Josy Miranda, Paula Santana, Thais Martins, Charles Ferreira, Iracilda Sampaio, Marcelo Vallinoto, Grazielle Evangelista Gomes

**Affiliations:** 1 Laboratório de Genética Aplicada, Instituto de Estudos Costeiros, Universidade Federal do Pará, Bragança, Pará, Brazil; 2 Laboratório de Genética e Biologia Molecular, Instituto de Estudos Costeiros, Universidade Federal do Pará, Bragança, Pará, Brazil; 3 Laboratório de Evolução, Instituto de Estudos Costeiros, Universidade Federal do Pará, Bragança, Pará, Brazil; Università degli studi di Napoli Federico II, ITALY

## Abstract

Molecular genetic techniques are an effective monitoring tool, but high-quality DNA samples are usually required. In this study, we compared three different protocols of DNA extraction: NaCl (saline); phenol-chloroform and commercial kit (Promega)—from three biological tissues of five individuals of *Lutjanus purpureus* under two methods of storage. The evaluated items included DNA concentration and purity, processing time and cost, as well as the obtaining of functional sequences. The highest average values of DNA concentration were obtained using the saline procedure and the commercial kit. Pure DNA was only obtained using the saline protocol, evaluated by the ratio of 260/280. The saline and phenol-chloroform protocols were the least expensive methods. The commercial kit costs are counterbalanced by the short time required. The procedure based on phenol-chloroform presented the worst results regarding DNA yield and the time required to perform all steps. The saline and commercial kit protocols showed similar results concerning the amount and quality of extracted DNA. Therefore, the final choice should be based on the available financial resources and the available time for carrying out each procedure of DNA extraction.

## Introduction

Fisheries products stand out as one of the leading food sources for humans (~88% of global production), resulting in a consumption rate of 20.3 kg/year/person [1]. Besides providing the recommended amounts of protein in the diet, fish and seafood, in general, are also rich in essential lipids and micronutrients for human health, such as the high content of polyunsaturated fatty acids (e.g. omega 3) combined with relatively low amounts of calories [2]. Regular fish consumption benefits humans by decreasing the risks of cardiovascular diseases, inflammatory diseases like arthritis, and even the development of cancer [3, 4].

Because the commercialization and the demand in fisheries have increased [1], several processed products have become available in the market. Processed products present advantages, such as accessible transportation and conservation, high quality, and profitable sales [5]. However, many diagnostic morphological features of fish species are lost during the production of process fisheries products, thus favoring illegal practices of species substitution [6–8].

Since several cases of intentional or unintentional species replacement have been reported, the development of tools that assure the identity of commercialized fish is required, thus preventing the trade of mislabeled products [9]. In this sense, several studies have shown the genetics relevance to detecting substitutions and investigating the authenticity of processed products [7, 10–12], showing that molecular tools can assist in inspection practices, provide DNA-based certification of the marketed product, and ensure monitoring of the fishing industry [13, 14].

The advancement of molecular biology has been remarkable over the last decades. For instance, a single capillary sequencer can process around half a million DNA sequences per year [15]. As a result of this approach, DNA sequencing in large-scale projects has become a common practice in distinct fields of biology, such as forensic practices, diagnosis of pathogens, ecological studies, and detection of organisms in clinical, food, and environmental samples in general [16–18]. The DNA barcode tool, based on Sanger sequencing, has been the most widely used approach for seafood identification. However, more advanced approaches have been applied for this purpose, such as Next Generation Sequencing (NGS), Single Nucleotide Polymorphism (SNP’s) and Real-Time PCR [19]. Even though genetic analyses represent a powerful tool to mitigate mislabeling and assure the certification of fisheries products, the practice of molecular certification, associated with poor legislation policies, remains largely neglected by inspection agencies and the fisheries industry in Brazil.

An essential step in genetic and molecular biology studies is obtaining of high-quality genetic material. Once the quality of extracted genomic DNA is guaranteed, it can avoid unnecessary expenses of financial resources and time, thus favoring the development of successful molecular techniques, such as Polymerase Chain Reaction (PCR), sequencing and molecular cloning [20].

Ever since the description of the first method of DNA extraction developed by Friedrich Miescher in 1869, several procedures for DNA extraction samples have been developed and widely used in the forensics and identification of biological products. These methods became particularly popular when specialized companies started producing commercial kits for DNA extraction [21]. In general, the distinct methods of isolating DNA share some common steps: lysis (break of plasmatic membrane), purification (removal of biological and chemical contaminants), and DNA recovery [22]. Nonetheless, it should be pointed out that a single method is not compatible with all types of samples (biological tissue). Therefore, the DNA extraction method selection relies on the sample origin, the purpose of the analysis, and the final targeted product [23].

Since efficient and optimized methods to obtain genomic DNA of satisfactory quality and integrity are required for molecular authentication of fish products, we compared three commonly used protocols of DNA extraction from three biological tissues conserved at distinct conditions: (1) extraction using saline solution (NaCl) [24]; (2) the phenol-chloroform-isoamyl alcohol method [25]; and (3) the commercial Wizard® Genomic kit (Promega). The present work aims to evaluate aspects related to DNA concentration and purity, the time of processing the samples according to each biological tissue, and the financial costs of each method. Based on these variables and considering the type of fish tissue and extraction methods, the most suitable DNA extraction protocol to successfully used in the fisheries industry or for the certification of processed fish products is presented.

## Materials and methods

All relevant data are within the paper and its Supporting Information files.

### Schematic overview of the experimental program

**Fig 1:**
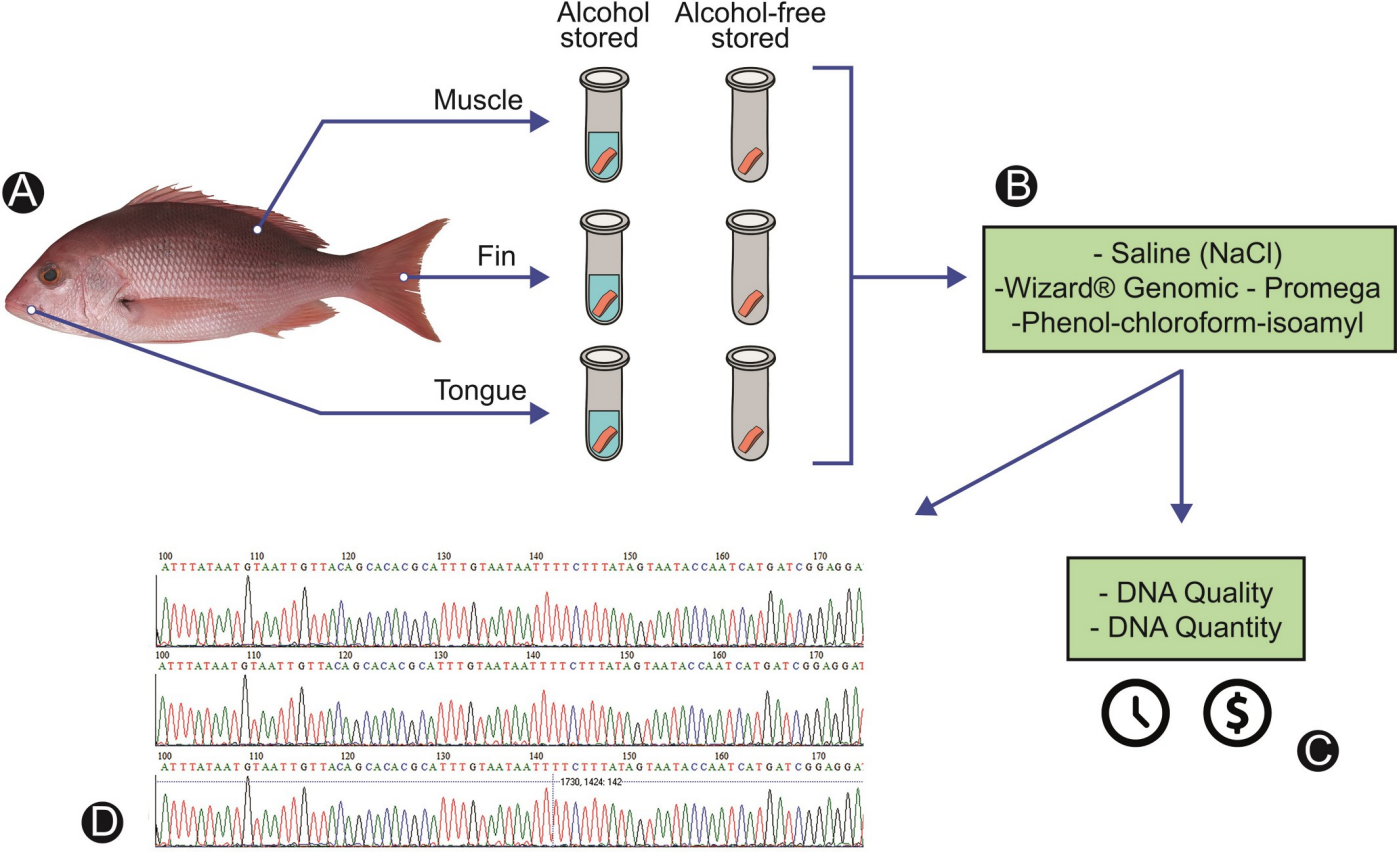
Flow diagram summarizing the experimental design. A: Species selection and tissue removal and storage conditions. B: DNA extraction with the three compared protocols. C: Analyzed variables, quality, and quantity of extracted DNA, costs, and time for sample processing according to the three extraction protocols. D: Verification of functional sequences from DNA barcode.

Fig 1 shows a schematic representation of the experimental design, showing the main steps for the accomplishment of this work, from the selection of the species and removal of the biological tissues to the observation of the different variables tested for each extraction protocol. We extracted three different types of tissue and used two forms of storage (Fig 1A) to observe a possible variation in the quality of the extracted DNA. In order to standardize the DNA extraction protocol for the molecular certification chain of processed fish, three methods were compared (Fig 1B), as well as the cost, time to process the samples, quality and quantity of extracted DNA (Fig 1C) and generation of functional sequences with the DNA barcode tool (Fig 1D).

### Target species and selection of biological tissues

The selected species was the red snapper *Lutjanus purpureus* (Poey, 1866) (Lutjanidae), one of the most exploited fish species from the northern coast of Brazil [26, 27] and highly commercialized as fillets or frozen specimens for national and international trade. The samples comprised five individuals of red snapper obtained from a fisheries port in Bragança (the northeastern region of the state of Pará, Brazil) in July 2021. This region is Brazil’s leading trade center of red snappers, providing processed fisheries products for several markets worldwide, such as the Caribbean, USA, and Europe [28]. The specimens were taxonomically identified using the specialized literature [29–31], and tissue samples were obtained at the landing points. Ethical approval was not required for the animal study because the animals were acquired at the fisheries port and were already dead.

The tissue samples included pieces of muscle, tongue, and caudal fins stored at frozen conditions (alcohol-free) or in 90% ethanol to be tested using the previously mentioned three protocols of DNA extraction. All samples were maintained at -20°C as part of the tissue bank of Lutjanidae in the Laboratório de Genética Aplicada (LAGA), Instituto de Estudos Costeiros (IECOS), at Universidade Federal do Pará (UFPA)/Bragança up to the moment of DNA extraction.

### Obtaining the genetic material: Methods of DNA extraction

Genomic DNA was extracted using three protocols from three tissues of five samples of *L*. *purpureus* stored under the abovementioned conditions. Therefore, 30 distinct assays were performed for each DNA extraction method, totaling 90 assays. The details of each DNA extraction procedure described below.

### DNA extraction using saline solution (NaCl)

The protocol reported by [24] was carried out with slight modifications. Firstly, 20 mg of macerated tissue was homogenized into 400 μL of lysis solution (6.5 mL of Tris-HCL, 2M pH 9.0; 10 mL of 0.1M EDTA pH 8.0; and 6 mL of 20% SDS) and 5 μL of RNAse (10 mg/mL). Then, the mixture was homogenized in a vortex for 10s and incubated at 37°C for 30 min. Afterward, 10 μL of proteinase K (10 mg/mL) was added, followed by incubation at 55°C for 3h. After checking that the tissue was completely dissolved, 350 μL of NaCl (5M in distilled water) was added to each sample. The samples were vortexed again for 10s, then frozen for 1h and centrifuged for 30 min at 10,000 rpm. The supernatant material (600 μL) was transferred to a new tube containing 600 μL of 100% isopropanol and centrifuged at 10,000 rpm for 15 min. The supernatant was discarded by tube inversion, 500 μL of refrigerated ethanol (70%) was added to the DNA pellet, and the tube was centrifuged for 15 min at 10,000 rpm. The liquid was discarded by tube inversion, and the tube was left open at 37°C for 15 min until the evaporation of alcohol residues was completed. The DNA was hydrated in a volume of 35 μL of ultrapure water at room temperature for 12 h and then stored at -20°C.

### DNA extraction using the commercial Wizard® Genomic kit (Promega)

Initially, 60 μL of 0.1M EDTA solution (pH 8.0) and 250 μL of Nuclei Lysis Solution were prepared in a falcon tube (15 mL) for each sample. This solution remained in the refrigerator for approximately 5 min. The macerated tissue (approximately 20 mg) was placed into 1.5 mL Eppendorf tubes containing 300 μL of the refrigerated EDTA/Nuclei Lysis Solution (one tube per sample). Subsequently, 5 μL of RNAse was added to each tube, vortexed for 10s, and then incubated at 37°C for 30 min. Then, 10 μL of proteinase K was added to each sample, followed by incubation at 55°C for 3 h. Afterward, 100 μL of Protein Precipitation Solution was added to each tube at room temperature, and the solution was vortexed at maximum speed for 10 s. The samples were kept at -20oC for 10 min and then centrifuged for 6 min at 13,000 rpm. The supernatant containing DNA was removed carefully and transferred to new tubes containing 300 μL of isopropanol. The solution was gently mixed by tube inversion and centrifuged for 10 min at 13,000 rpm. The supernatant was then discarded carefully. 300 μL of 70% ethanol was added at room temperature, inverted the tubes several times to wash the DNA, and then centrifuged the samples for 5 min at 13,000 rpm. The ethanol was carefully discarded by tube inversion. Then, the tubes were left open at 37°C for 15 min up to the complete evaporation of alcohol. Rehydration was performed using 35 μL of ultrapure water for 12 h. After this period, the extracted DNA products were stored at -20°C.

### DNA extraction using the phenol-chloroform-isoamyl alcohol

The protocol by [25] was performed with some modifications. About 20 mg of macerated tissue was placed into a 1.5 mL Eppendorf tube containing 600 μL of lysis buffer (Tris-HCL, 2 M pH 9.0; EDTA, 0.1 M pH 8.0; 20% SDS and distilled water). After adding 5 μL of RNAse, the solution was incubated at 37°C for 30 min. Then, 15 μL of Proteinase K was added, followed by incubation for 3h of incubation in a moist chamber at 55°C or until the tissue was completely dissolved. We carried out two washing steps in the lysed material. Firstly, we added 700 μL of phenol-chloroform-isoamyl alcohol (25:24:1 ratio), mixed the solution manually for 10 min to homogenize the mixture, and then centrifuged the samples at 10,000 rpm for 10 min. Afterward, the supernatant was removed and transferred to a new tube containing 700 μL of chloroform-isoamyl alcohol (24:1 ratio), which was again manually mixed for 10 min and centrifuged at 10,000 rpm for 10 min. The top layer of supernatant was carefully removed and transferred to another Eppendorf tube, followed by the addition of 100 μL of sodium acetate (AcNa 3 M pH 4.8) and homogenization. To precipitate the extracted DNA, 700 μL of isopropanol was added at room temperature, and the samples were stored at -20°C for 1h. The tubes containing the DNA pellets were then centrifuged for 10 min at 10,000 rpm, and the supernatant was carefully discarded. Afterward, 200 μL of 70% ethanol was added at -4°C, and the samples were centrifuged again for 5 min at 10,000 rpm. The supernatant was discarded, and the DNA pellet was dried at 37°C. Finally, the DNA was hydrated in 35 μL of ultrapure water for 12 h and then stored at -20°C.

### DNA quality: Visualization in agarose gel

The extracted DNA samples from each treatment were mixed in 3 μL of Gel red™ and Blue Juice (55% glycerol, 0.5 M EDTA, 0.1% bromphenol blue, 0.1% xylene cyanol) staining solution at a proportion of 1:1, followed by horizontal electrophoresis in 1% agarose gel at 70 V for 60 min. Then, the DNA products were visualized under UV light to evaluate the quality of the DNA samples. 2,8 kb ladder molecular size markers were used for orientation.

### Quantification and degree of purity in DNA samples

The quantification (ng/μL) and the degree of DNA purity were evaluated using a NanoDrop 2000 spectrophotometer (Thermo Scientific™). Usually, values between 1.7 to 2.1 for the A260/A280 ratio and from 1.8 to 2.2 for A260/A230 ratio are pure DNA samples [32–34]. Therefore, the same parameters were considered in the present work.

### Analyses of economic cost and time demanded for DNA extraction

The financial costs (in US$) required to each DNA extraction procedure were estimated for individual samples, including those related to consumables and reagents, based on average prices provided by supplier companies. The time required for DNA extraction was calculated, from tissue maceration to DNA hydration. The calculation was also performed for processing the samples by comparisons among the three protocols of DNA extraction. Ultimately, a price quotation out to determine the average cost necessary to visualize DNA samples in agarose gel.

### Statistical analysis

A two-way analysis of variance (ANOVA) was performed to compare the DNA concentration and purity (260/280 and 260/230) ratio values according to each protocol for storage method and tissue for all samples of *L*. *purpureus*. The statistical analyses were carried out using the software R 4.2.2 [35], using the *rstatix* package [36]. The Sidak method was used for post hoc multiple comparison analysis with *emmeans* package [37] and considering a 5% significance level (*p* < 0.05). Results are shown as mean and standard error (se) of the mean. The graphics were generated with the ggplot 2 package [38].

### Validation of DNA samples based on barcode COI sequences

Initially, four samples from four individuals of red snapper (*L*. *purpureus*) were tested for DNA barcode analyses during the experimental design of the present work. Muscle tissues were collected and stored in 90% alcohol, and total DNA was extracted considering the three abovementioned protocols.

The DNA extracted from such initial samples was evaluated only concerning quality via horizontal electrophoresis in 1% agarose gel followed by visualization under UV light. Afterward, Polymerase Chain Reaction (PCR) was carried out using the extracted DNA template to amplify a portion of the mitochondrial Cytochrome Oxidase, subunit I (COI) gene referred to as DNA barcode. The PCR primers and amplification conditions followed those reported by [39] and [40], respectively.

PCR products quality was evaluated in agarose gel as previously described and then purified in PEG (Polyethylene Glycol) 8000, according to [41], followed by dideoxyterminal sequencing [42] using Big Dye 3.1 kit (ABI Prism™ Dye Terminator Cycle Sequencing Ready Reaction—PE Thermo Fisher) according to the manufacturer’s instructions. Afterward, the precipitated products were read in an automatic capillary sequencer, model ABI 3500 XL (Thermo Fisher).

The barcode COI sequences were checked by inspection of electropherograms and compiled into a dataset using the software BioEdit v. 7.2.5 [43]. The automatic sequence alignment was performed using Clustal X [44], available in BioEdit v. 7.2.5 [43].

The aligned sequences were submitted to the public platforms GenBank NCBI (National Center for Biotechnology Information—http://www.ncbi.nlm.nih.gov) [45], using the BLAST search tool (Basic Local Alignment Search Tool), and BOLD Systems (Barcoding of Life Database—http://www.barcodinglife.org) [46] to confirm the taxonomic identification of samples. To improve the molecular identification analysis, public sequences available for Lutjanidae species in Brazil were included [40, 47, 48]. Sequences of *Genyatremus luteus* (Haemulidae) were used as an outgroup. The access codes of the generated sequences and the public dataset are available in S1 Table.

Once the final dataset was compiled, we generated a Neighbor-Joining phylogenetic tree using the Kimura-2-parameter (K2P) evolutionary model [49] with 1,000 pseudoreplicates of bootstrap [50] in the software MEGA 11 v11.0.11 [51]. The presence of stop codons was also evaluated using the same software. The Neighbor-Joining tree was visualized in FigTree, v1.4.4 [52] and edited in InkScape v0.92.4 (https://www.inkscape.org).

## Results

### Visualization in agarose gel

**Fig 2:**
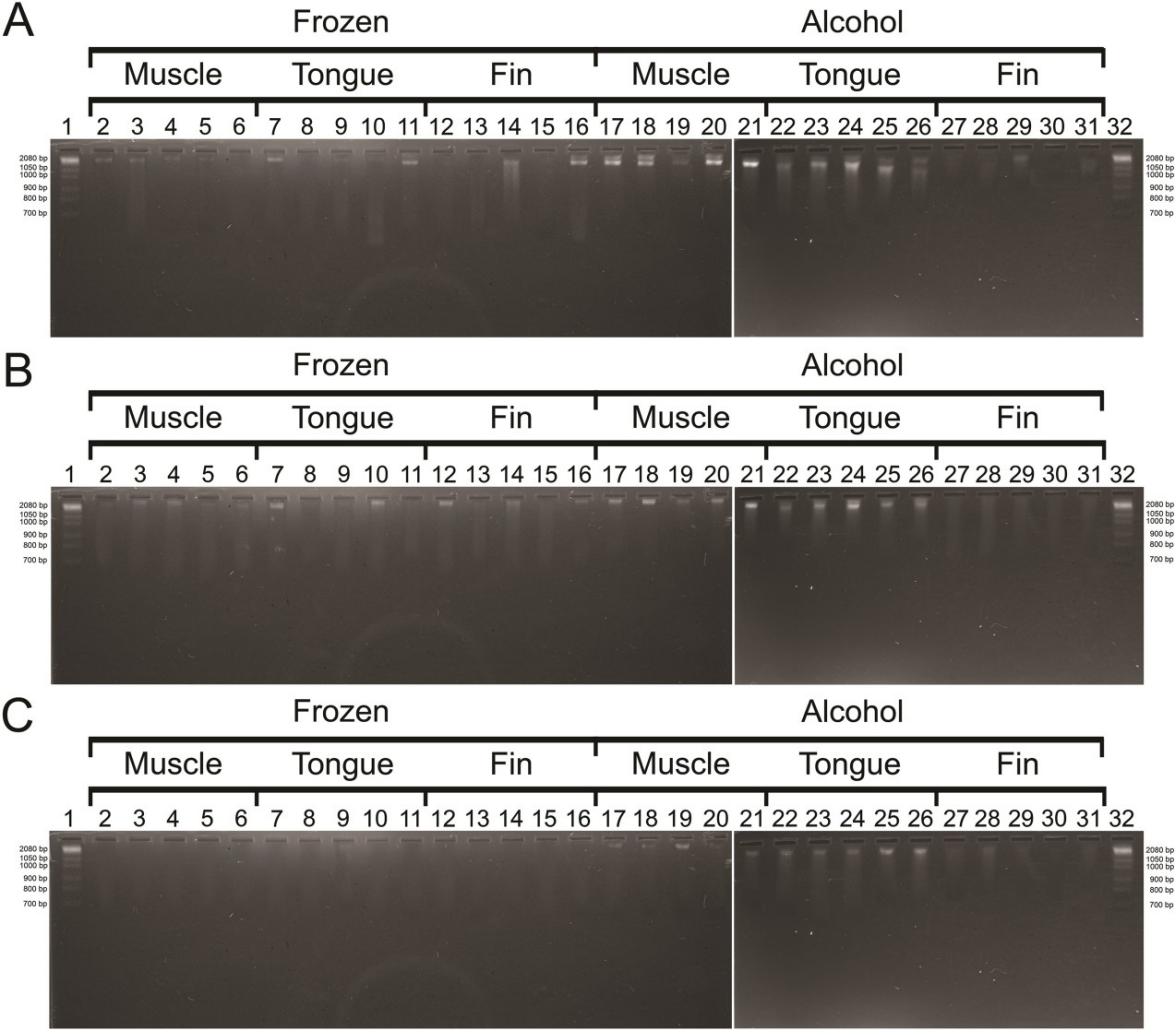
Electrophoresis of DNA isolated from *L*. *purpureus* by three different methods. A: saline (NaCl) protocol, B: Wizard® Genomic kit—Promega, C: Phenol-chloroform-isoamyl alcohol. Lanes 1 and 32: 2,8 kb ladder molecular size markers. A white line indicates that lanes 20 and 21 are of the same gel.

The results of the analyses of DNA samples visualized in agarose gels (Fig 2) were similar between the three DNA extraction methods. Well-preserved DNA, with little evidence of degradation, was obtained in most of the samples extracted using all the three methods, being particularly successful in samples 15 to 20 (Fig 2, lines 16 to 21). However, the phenol-chloroform protocol yielded low amounts of extracted DNA in some samples (e.g. 1 to 14, lines 2 to 15) (Fig 2C).

### Concentration and purity of DNA samples

**Fig 3:**
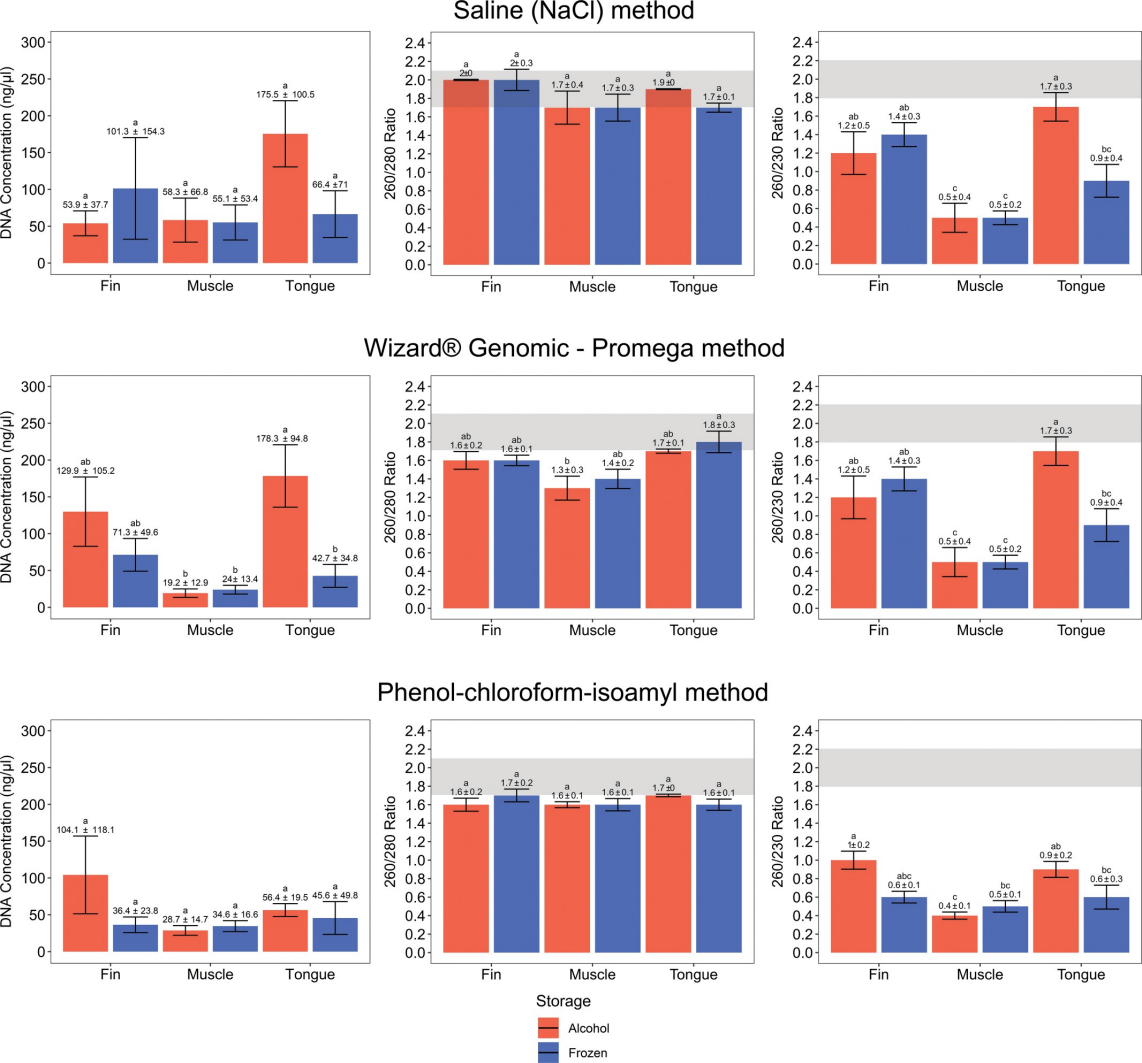
DNA concentration, 260/280 and 260/230 purity ratio for the three protocols of DNA extraction according to tissue type and storage conditions. Gray shaded areas indicate the ratio of purity values considered satisfactory for extracted DNA. Error bars indicate standard deviations for the five independent replicates. Different letters indicate significant differences (*p* <0.05) from each comparative analysis.

The values of DNA quantification and 260/280 and 260/230 purity ratios for the 30 assays for each method of DNA isolation are shown in S2 Table. The mean, minimum, maximum, and standard error (se) values of DNA quantification and purity ratios for each method of DNA extraction along with the summary of the statistical analyses are shown in S1 File. Considering the five samples of *L*. *purpureus* used as biological replicates, a variation in DNA concentration was observed according to each tissue and storage condition (Fig 3). Generally, saline solution and commercial kit methods resulted in a higher DNA concentration than the phenol-chloroform method. The highest average value (~170 ng/μL) of extracted DNA was obtained using a tongue stored in alcohol for both saline and commercial kit protocols. In contrast, using the phenol-chloroform method, the fin tissue provided the highest mean value of DNA concentration (~100 ng/μL).

By comparing the DNA concentration from each DNA extraction method, the saline and phenol-chloroform methods showed similar results, i.e., no significant difference in the extracted DNA concentration was observed according to tissue or storage condition. However, the mean DNA concentration using the commercial kit differed, with higher values for the DNA samples extracted from the tongue stored in alcohol than in muscle (either frozen or stored in alcohol) or frozen tongue tissue. Even though the highest DNA concentration was reported in samples extracted using saline and the commercial kit, some tissues stored at different conditions presented values close to zero. For instance, muscle tissue stored in alcohol and frozen, frozen fins, tongue in alcohol and frozen (in the case of saline protocol), and muscle in alcohol and frozen tongue (commercial kit). In contrast, the phenol-chloroform method invariably yielded minimum values of DNA concentration higher than 10 ng/μL for all tissues and storage conditions (S2 Table).

The 260/280 and 260/230 DNA purity ratios diverged among DNA extraction methods, resulting in differences in the classification of DNA samples as pure. Using the saline method, all mean values for the 260/280 ratio varied between 1.7 and 2.1 without significant difference. For the 260/230 ratio, average values above 2.1 were observed for frozen muscle and fin tissues, but with no significant differences. When the commercial kit was evaluated, the 260/280 ratio in DNA extracted from frozen/alcohol-stored muscle presented average values below 1.7, regarded as non-pure samples. The 260/230 ratio showed all average values being below 1.8. Once more, the muscle tissue yielded the lowest DNA purity levels (~0.5) in both storage conditions. In the case of the phenol-chloroform method, the 260/280 ratio presented all three tissues and both storage methods with relative values. Nonetheless, most mean values were below 1.7, where only the tongue in alcohol had values compatible with pure extracted DNA samples. Similar to the commercial kit pattern, the 260/230 ratio mean values in DNA samples extracted following the phenol-chloroform method were below 1.8 (S2 Table).

### Economic costs and time demanded for DNA extraction

A comparative evaluation of the financial costs associated with each DNA extraction method is shown in Table 1, assuming the quoted values of reagents and consumables used to extract DNA of individual samples in each method at the time this study was performed. The saline and phenol-chloroform methods were the least expensive ones. The expenses related to consumables and reagents in either saline or phenol-chloroform methods represent about 45.5% and 42.5% of the costs required for DNA extraction using the commercial Wizard® Genomic kit (Promega), respectively. The costs of both saline and phenol-chloroform protocols are quite similar, with a slight difference of 10 cents ($), mainly because the phenol-chloroform method requires more plastic material.

**Table 1:** Comparison of the costs (in US dollars) required to isolate DNA per sample according to the three DNA extraction methods.

	Procedure		
	Saline (NaCl)	Wizard® Genomic (Promega)	Phenol-chloroform-isoamyl
**Plastic material**	0.85	0.84	0.94
**Reagents**	0.93	2.43	0.94
**Total**	1.78	3.27	1.88

The costs of the genomic Wizard®kit (Promega) was counterbalanced by the reduction in time of processing the DNA extraction for a single sample (about 4 h and 32 min). The time required to carry out DNA extraction using the saline protocol was equal to 6h, while the phenol-chloroform method requires up to 6 h and 12 min of laboratory work.

### Identification of COI sequences

**Fig 4:**
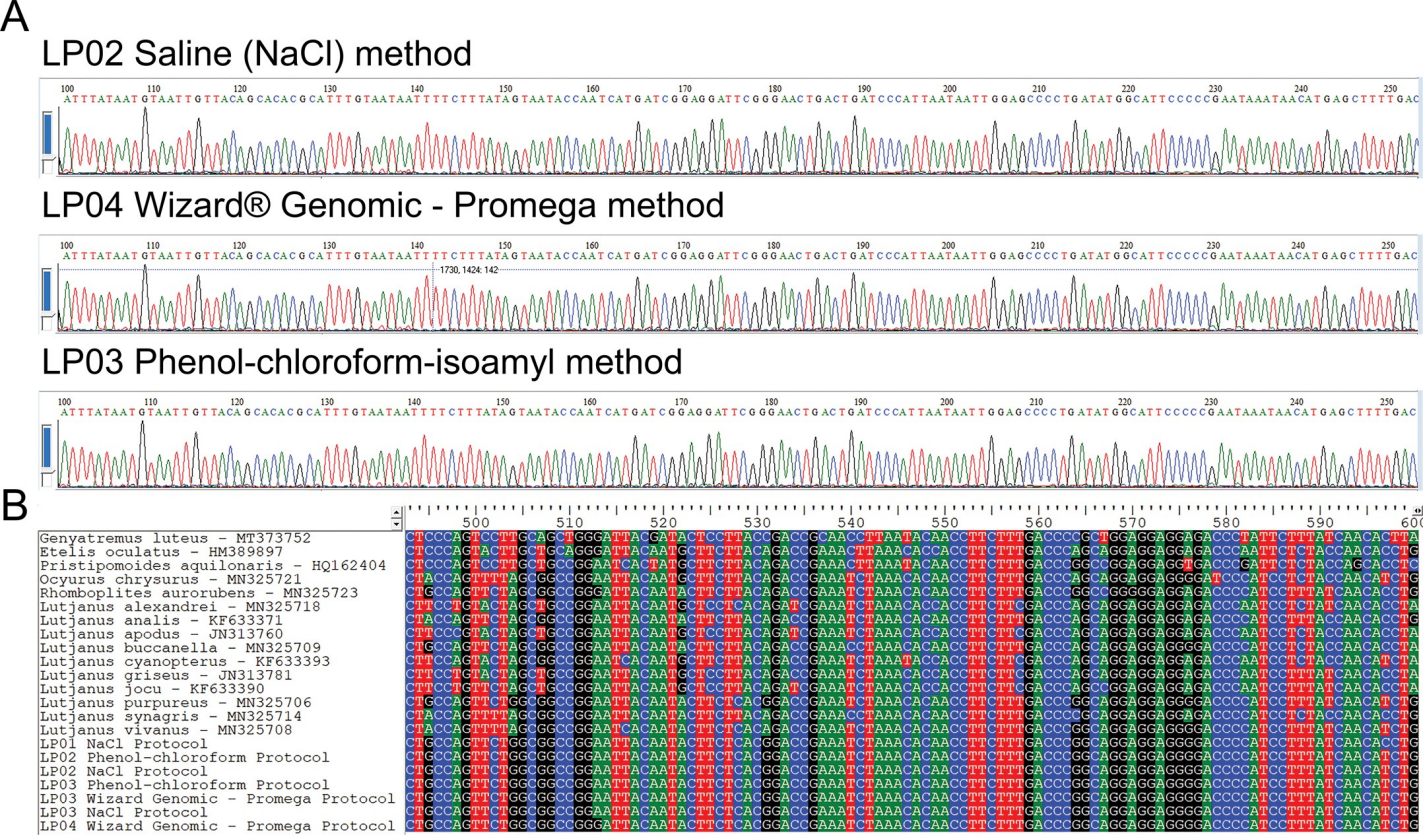
Portions of COI sequences from *L*. *purpureus* according to each DNA extraction method. LP: *Lutjanus purpureus*. A: Electropherograms in three samples of red snapper. B: Sequence dataset encompassing the seven COI sequences in a pilot essay with *L*. *purpureus* and other Lutjanidae representatives from the Brazilian coast. The species *Genyatremus luteus* (Haemulidae) was used as an outgroup.

**Fig 5:**
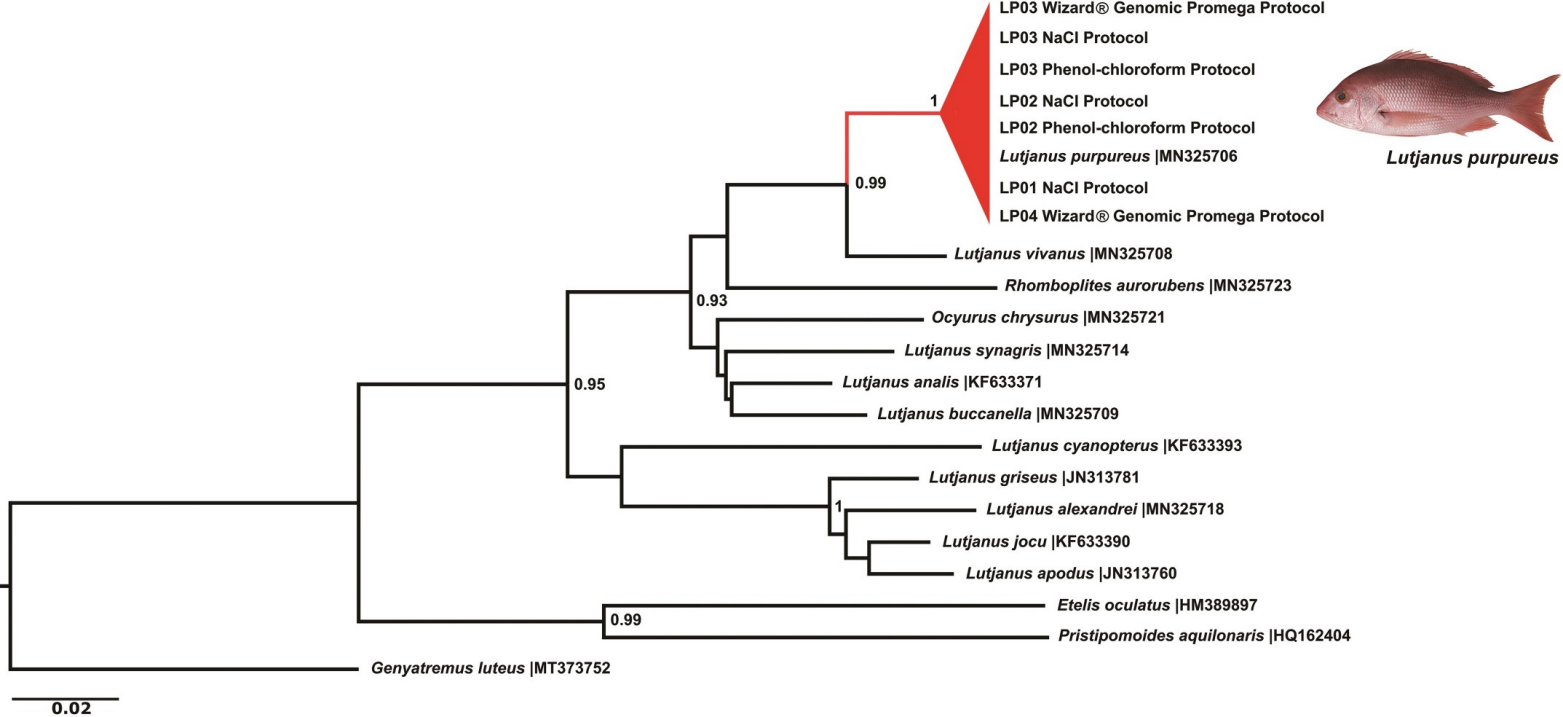
The Neighbor-Joining tree obtained from the pilot essay with COI sequences of *L*. *purpureus* obtained from three DNA methods and other Lutjanidae species from the Brazilian coast. LP: *Lutjanus purpureus*. The species *Genyatremus luteus* (Haemulidae) was used as an outgroup.

Based on the three DNA extraction methods, the fragments of 600 bp of the COI gene used as DNA barcodes were obtained for seven replicates of *L*. *purpureus* (S1 Table). All electropherograms were visually inspected for a qualitative assessment of COI sequences. The nucleotides could be unequivocally confirmed according to their respective fluorescent peaks, proper spacing, and signal intensity (Fig 4A), regardless of the DNA extraction protocol. No insertions, deletions, or stop codons were observed in the obtained sequences (Fig 4B). After incorporating COI sequences available in public databases, the Neighbor-Joining tree confirmed the morphological identification of individuals as *L*. *purpureus* by forming a clade clustering intraspecific sequences, including Lutjanidae family representatives from the Brazilian coast (Fig 5).

## Discussion

### Utilization of molecular genetics in fisheries

The use of molecular genetics as a tool in forensics and for the certification of commercial products has proven to be highly efficient in solving fraud cases or substituting seafood products [7, 53]. For instance, several reports based on genetic data of commercialized fish products have identified endangered species [7, 54], providing helpful information about the illegal trade of species threatened with extinction [55].

A certified product guarantees the transparency for consumers, as well as food safety and traceability of exploited species, being helpful to the effective management of fisheries resources [56, 57]. Compared to other biotechnology tools for fish identification and traceability (e.g. geochemical and biochemical methods), the procedures based on molecular genetics still present some drawbacks, such as high costs and the time spent to analyze the samples [58]. However, recent advances in genetic analyses have optimized this process, reducing both the financial costs and the highly specialized labor required [59]. Therefore, DNA-based methods represent a highly recommended tool for inspection and certification analysis, since they are more selective, sensitive and reliable than other methodologies, thus being particularly useful to fisheries industry [60].

A variety of protocols for DNA extraction have been compared to evaluate the most suitable method for a particular goal and type of sample, including different phyla and groups [61, 62], food products [63], fecal samples [64], blood traces [65] and ancient samples [66]. [67] recently compared different DNA extraction methods for seafood products. However, the methods analyzed may not yet be a reality for the fishing industries of developing countries, given the lower purchasing power of these countries to acquire more modern DNA extraction kits. Unlike [67], we used the published saline and phenol-chloroform methods that are still commonly cited in the scientific literature [68]. Furthermore, we added some variables that could alter the quality and yield of the extracted DNA, such as three different biological tissues (muscle, tongue, and fin) and two different forms of storage (only frozen without alcohol and frozen in 90% ethanol).

### Concentration and quality of DNA samples

The amount and the quality (integrity and purity) of extracted DNA are critical factors to their successful application and reproducibility in molecular genetics [20]. As the individuals of *L*. *purpureus* analyzed were acquired directly at the time of landing from a commercial fleet, with no intention of being the object of scientific study, we did not obtain information on the time elapsed between capture and collection of tissues for storage, which may influence DNA degradation. Therefore, we recommend observing this information in the future studies when available. In this sense, the method based on saline solution and the commercial Wizard® Genomic kit (Promega) yielded the highest integrity (Fig 2) and the highest concentration (Fig 3) values in DNA samples. The commercial kit (Promega) presented the best yields in the study by [67], due to the larger volume of biological tissue and the more significant amount of Proteinase K used concerning the other methods analyzed. However, when checking efficiency (i.e. ng of DNA extracted per mg of tissue) other protocols proved to be better, such as the Chelex protocol [67]. In contrast, our standardized the amount of biological tissue (20 mg) and Proteinase K (10 μL) for the saline methods and the commercial kit, eliminating these factors from the results.

Based on the visualization in agarose gel (Fig 2), the best DNA bands profile considering their integrity and high molecular weight was observed in the protocols based on saline and the commercial kit despite some variation according to tissue and storage condition (Fig 2A and 2B). On the other hand, the phenol-chloroform method yielded many samples with low DNA concentration (Fig 2C).

In general, the phenol-chloroform protocol, a traditional DNA extraction method, had the worst results concerning the amount of extracted DNA (Fig 3), differing from other studies that reported satisfactory results using the same procedure [69–71]. The study by [68] demonstrated that the phenol-chloroform method gave the best results for extracted DNA, regardless of the type of product analyzed, such as fresh or processed tuna. [72] analyzing tissues from *Lutjanus jocu*, *Epinephelus itajara* and *Centropomus parallelus* obtained an overall mean of 2547.6 ng/μl for liver, 1809.5 ng/μl for muscle, and 524.4 ng/μl for fin, a result contrary to what we found in the present study. Even if the objective is not to verify the efficiency of the method, different studies have used the phenol-chloroform method to generate functional sequences. [73] analyzed hypervariable sequences for phylogeography study of *Lutjanus analis* and *L*. *jocu*, [74] produced multiplex PCR for the identification of the shark *Isogomphodon oxyrhynchus*, [75] evaluated the diversity of Neotropical anurans using DNA barcodes, [76] made the molecular phylogenetic inference of Primates *Alouatta*, and [77] identified the commercialization of the critically endangered species *Pristis perotteti*. However, despite these examples and the good acceptance by the scientific literature, the best concentration of extracted DNA does not always come from the phenol-chloroform method [69]. Our results suggest that the phenol-chloroform method can be readily replaced by other less harmful methods for human health and the environment. Phenol and chloroform are highly toxic organic solvents that cause skin burns, hepatotoxicity, nephrotoxicity, and carcinogenesis [78–80].

Visualizing DNA samples in agarose gel provides useful qualitative information based on the presence and intensity of bands after electrophoresis. The estimates using a spectrophotometer assure the reliability of DNA concentration and yield, removing the observer’s subjectivity. This is an essential aspect for the certification of processed products, besides saving the time spent in analyses by disregarding the preparation of agarose gel and subsequent electrophoresis run (~40 min in the present study). In addition, costs are also reduced since it would be necessary to be around $1.21 (US dollars) to visualize an individual sample on an agarose gel according to the price quotation. Of course, there is an initial investment in acquiring a spectrophotometer (the model used in this study was evaluated at $8,725.00 on average). Nonetheless, this value is offset by the continuous utilization of this equipment, such as that predicted in fisheries industries, ensuring fewer expenses over time.

Concerning the storage conditions, the best results were observed in samples preserved in alcohol rather than just frozen using the three protocols of DNA extraction (Figs 2 and 3). By preserving tissues in alcohol, bacterial proliferation and the degradation of nucleic acids by the activity of nucleases are avoided [81, 82]. Although time is a critical factor in the fisheries industry, the preparation of tissues stored in alcohol increases the chances of obtaining the best results, even in cases when DNA is extracted shortly after fish landing.

Most molecular studies rely on pieces of muscle for DNA extraction because it is a soft tissue of easy maceration, favorable to digestion by proteinase K in short period of time [83]. However, the highest mean values in DNA concentration were observed in tongue samples for the protocols of DNA extraction based on saline and commercial kit and fins for the phenol-chloroform method. From a general point of view, the use of tongue and fins for DNA extraction preserves the external morphology of fishes, thus avoiding depreciation in trade markets without compromising their certification, representing a significant added value, particularly in exported products. In addition, using fin or tongue tissues is also recommended to obtain DNA samples from museum specimens [83] that cannot be damaged.

### DNA purity

DNA purity ratios between 1.7–2.1 (A260/A280) and 1.8–2.2 (A260/A230) are regarded as indicators of pure samples of extracted DNA concerning the presence of organic matter and solvent residues [32–34]. When these residues are present in DNA samples, the following steps of genetic analyses, such as PCR, can be threatened since they act as these inhibitors, thus being a critical aspect to evaluate during procedures of DNA extraction [84].

The purity of DNA samples extracted using the three methods of DNA extraction in this study was variable (S2 Table). Regarding the 260/280 ratio, the saline method was the only method yielding mean values between 1.7 and 2.1 (S2 Table). The Wizard® Genomic kit (Promega) also resulted in satisfactory purity levels, with values below 1.7 observed only in frozen and alcohol-stored muscles (S2 Table). On the other hand, all mean purity values obtained using the phenol-chloroform method were below 1.7 (S2 Table). Usually, the presence of proteins and residues such as phenol and ethanol are implied in the reduced values in the 260/280 ratio [85]. Most likely, these contaminants were present in the extracted DNA samples with low purity values, as reported using the Wizard® Genomic kit (Promega) and the phenol-chloroform method.

Despite the average values being close to the parameters referred to as pure DNA, some samples presented values much higher than the expected (2.1). Since the contamination by RNA can increase the 260/280 ratio [86], several protocols of DNA extraction require the utilization of RNAse to degrade RNA, as recommended by [33] when using the saline method of DNA extraction. Therefore, the utilization of an RNAse digestion step would decrease the high values observed (>2.1) in the 260/280 ratio (S2 Table).

Unlike the 260/280 ratio, the saline method of DNA extraction resulted in mean values of 260/230 ratio above 2.1 in both frozen fin and muscle tissues. At the same time, the phenol-chloroform and the commercial kit yielded mean 260/230 ratio values below 1.8 (S2 Table). In the latter, some DNA samples with values inferior to 0.5 were reported, indicating a high degree of contamination. The low values in the 260/230 ratio indicate the presence of contaminants coextracted with DNA molecules, such as phenol and guanidine [87]. Therefore, despite the variation in the purity degree according to each method and tissue, the analyzed variables indicated that the saline method is the most suitable for DNA extraction (S2 Table).

### Comparative efficiency based on costs, time, and technical requirements

The development of commercial kits made it possible to shorten the time spent in DNA extraction compared to other published protocols, such as those based on saline solution and phenol-chloroform. Most DNA extraction kits require ready-to-use reagents, reducing the time in preparing the material and demanding a low number of operational steps, making the whole process simple and easily performed even by an untrained technician. On the other hand, while commercial kits are time-saving and straightforward methods, their utilization increases the cost associated with the extraction of an individual sample, representing an increase of 45.6% and 42.5% for the saline or the phenol-chloroform methods, respectively (Table 1).

Therefore, both the saline and phenol-chloroform methods were the most economical, with a 5.61% difference in cost between them(Table 1). Even though they demand longer steps of incubation, centrifugation, and tube transfers that increase the time spent in DNA extraction. Moreover, the phenol-chloroform method is highly harmful to health due to the toxic characteristics of the organic compounds used [78–80], requiring extreme caution and the utilization of a fume hood to prevent the inhalation of these compounds. Also, some plastic tubes cracked during centrifugation processes in the phenol-chloroform method, related to the presence of these compounds combined with high pressure. To prevent these incidents, it was necessary to use more resistant tubes, which increased the costs of material used per extracted DNA sample (Table 1).

The results of the saline method yielded similar to those obtained using the Wizard® Genomic kit (Promega) concerning the quantity and quality of extracted DNA samples (Fig 3), besides being the least expensive method among the three methods tested (Table 1). Therefore, fisheries industries witch limited infrastructure or reduced budgets rely on methods based on saline solution to obtain high-quality DNA samples while saving financial resources.

### Validation of barcode COI sequences

The three DNA extraction methods were tested in a pilot study of DNA barcodes using muscle tissue from seven individuals of red snapper (*L*. *purpureus*). Genetic analyses can only be performed based on functional DNA fragments validated by sequencing (Fig 4). In this sense, an effective DNA extraction process is essential to ensure the success of the following steps of molecular techniques, like PCR and sequencing [16]. The successful amplification via PCR of the barcode COI region showed that the three methods effectively extracted DNA in sufficient quantity, integrity, and purity to perform sequencing a 600 bp fragment of the COI gene (Fig 4).

The validation test resulted in high-quality sequences since all nucleotides could be unequivocally read (Fig 4), applicable to several studies and approaches. For instance, the sequences used in this pilot assay resulted in the correct identification of sampled species and the reliable discrimination of Lutjanidae in the Neighbor-Joining tree, regardless of the tested extraction method (Fig 5), confirming their utilization as efficient authentication tools.

Frauds in the commercialization of processed products have been reported in Brazil for several species of fish, such as in marine and freshwater groups of catfishes and drums [7, 8, 88], besides the red snapper *L*. *purpureus* [10]. These reports highlight the urgent need to develop standardized DNA-based methods of authentication and certification of fish to ensure reliable and efficient inspection of traded fish products.

DNA amplification and sequencing success demonstrate that the three DNA extraction methods can be used in fisheries. Nonetheless, other variables must be considered, such as time and costs, the availability of biological tissues, infrastructure, and possible toxic effects related to each method. After ensuring good yield and quality DNA in the initial extraction step, the following steps of molecular techniques should also be standardized and optimized for better utilization by the routine fishing industry. The COIBar-RFLP technique, which combines the Barcode region of the COI gene with the RFLP (Restriction Fragment Length Polymorphism) method [89, 90], and Multiplex PCR protocols [91] are some examples of methods capable of identifying and revealing fraud in food products without the need for sequencing. Therefore, decreasing costs and sample processing eliminates the need for specific skills and knowledge on the operator’s part.

## Conclusions

In this study, we compared three DNA extraction methods commonly used in scientific reports and by regulatory agencies in several countries. Considering the worst results, risks to human health, and the environment related to the use of phenol-chloroform and the longer time required to perform all the steps of the method does not make it interesting for routine use in the fishing industry. We recommend that this method be replaced by others, such as those based on the saline solution or commercial kits. Both saline protocol and Wizard® Genomic kit (Promega) provided similar results concerning the quantity and quality of extracted DNA. Therefore, the final choice would depend on the available financial resources and time for carrying out each procedure. While the Wizard® Genomic kit (Promega) requires less time to isolate DNA samples, the costs of reagents increase considerably. On the other hand, the protocol based on the saline solution is less expensive even though it requires a relatively long period to be performed, being suitable for small fisheries industries. Concerning the biological tissue and storage form, we propose tongue and fin, and storage in alcohol as indicated for best results in concentration and purity of extracted DNA.

Finally, we recommend that new comparisons between extraction protocols be performed in future studies, inserting new commercial kits and maintaining the published classical protocols. These protocols should also be tested on other fishery products, such as cephalopods, mollusks, and crustaceans, as well as on tissues stored in other conditions.

## Supporting information

S1 Table Sequences used for the identification and genetic analysis of samples of *Lutjanus purpureus* in comparative analyses of DNA extraction methods. LP: *Lutjanus purpureus*.
(DOCX)

S2 Table DNA quantification and purity ratio according to each tested protocol of DNA extraction in samples of *Lutjanus purpureus*. (DOCX)

S1 File Summary of statistical analyses with mean, minimum, maximum and standard error (se) values of DNA quantification and purity ratios for each DNA extraction method in samples of *Lutjanus purpureus*. (DOCX)
