# Supplementary material for: Quality analysis of genomic DNA and authentication of fisheries products based on distinct methods of DNA extraction
Source: PLoS One. 2023 Feb 28;18(2):e0282369. doi: 10.1371/journal.pone.0282369 (PMC9974130; doi:10.1371/journal.pone.0282369)
Supplement: S1 Table — LP: Lutjanus purpureus. (DOCX) [file pone.0282369.s001.docx]

**Table S1.** Sequences used for the identification and genetic analysis of samples of *Lutjanus purpureus* in comparative analyses of DNA extraction methods. LP: *Lutjanus purpureus*.

| **Species** | **Access Code** | **Platform** | **Reference** | **Identification** |
| --- | --- | --- | --- | --- |
| *Lutjanus purpureus* | OP866759 | NCBI | Present study | LP01 Saline (NaCl) method |
| *Lutjanus purpureus* | OP866760 | NCBI | Present study | LP02 Phenol-chloroform-isoamyl method |
| *Lutjanus purpureus* | OP866761 | NCBI | Present study | LP02 Saline (NaCl) method |
| *Lutjanus purpureus* | OP866762 | NCBI | Present study | LP03 Phenol-chloroform-isoamyl method |
| *Lutjanus purpureus* | OP866763 | NCBI | Present study | LP03 Wizard® Genomic - Promega method |
| *Lutjanus purpureus* | OP866764 | NCBI | Present study | LP03 Saline (NaCl) method |
| *Lutjanus purpureus* | OP866765 | NCBI | Present study | LP04 Wizard® Genomic - Promega method |
| *Etelis oculatus* | HM389897 | NCBI | Unavailable | - |
| *Lutjanus alexandrei* | MN325718 | NCBI | Veneza et al. (2019) | - |
| *Lutjanus analis* | KF633371 | NCBI | Veneza et al. (2014) | - |
| *Lutjanus apodus* | JN313760 | NCBI | Unavailable | - |
| *Lutjanus buccanella* | MN325709 | NCBI | Veneza et al. (2019) | - |
| *Lutjanus cyanopterus* | KF633393 | NCBI | Veneza et al. (2014) | - |
| *Lutjanus griseus* | JN313781 | NCBI | Unavailable | - |
| *Lutjanus jocu* | KF633390 | NCBI | Veneza et al. (2014) | - |
| *Lutjanus purpureus* | MN325706 | NCBI | Veneza et al. (2019) | - |
| *Lutjanus synagris* | MN325714 | NCBI | Veneza et al. (2019) | - |
| *Lutjanus vivanus* | MN325708 | NCBI | Veneza et al. (2019) | - |
| *Ocyurus chrysurus* | MN325721 | NCBI | Veneza et al. (2019) | - |
| *Pristipomoides aquilonaris* | HQ162404 | NCBI | Gold et al. (2011) | - |
| *Rhomboplites aurorubens* | MN325723 | NCBI | Veneza et al. (2019) | - |
| *Genyatremus luteus* | MT373752 | NCBI | Unavailable | - |
