# Supplementary material for: Quality analysis of genomic DNA and authentication of fisheries products based on distinct methods of DNA extraction
Source: PLoS One. 2023 Feb 28;18(2):e0282369. doi: 10.1371/journal.pone.0282369 (PMC9974130; doi:10.1371/journal.pone.0282369)
Supplement: S2 Table — (DOCX) [file pone.0282369.s002.docx]

**Table S2.** DNA quantification and purity ratio according to each tested protocol of DNA extraction in samples of *Lutjanus purpureus*.

| **Sample** | **Tissue** | **Storage** | **Method** | **DNA concentration (ng/µl)** | **A260/A280 ratio** | **A260/A230 ratio** |
| --- | --- | --- | --- | --- | --- | --- |
| 1 | Muscle | Frozen | Saline (NaCl) | 16.9 | 1.96 | 1.47 |
|  |  |  | Wizard® Genomic - Promega | 39.3 | 1.51 | 0.57 |
|  |  |  | Phenol-chloroform-isoamyl | 18.3 | 1.54 | 0.52 |
| 2 |  |  | Saline (NaCl) | 46.9 | 1.97 | 2.04 |
|  |  |  | Wizard® Genomic - Promega | 23.8 | 1.47 | 0.44 |
|  |  |  | Phenol-chloroform-isoamyl | 31.7 | 1.4 | 0.53 |
| 3 |  |  | Saline (NaCl) | 2.6 | 1.65 | 15.21 |
|  |  |  | Wizard® Genomic - Promega | 34.9 | 1.65 | 0.73 |
|  |  |  | Phenol-chloroform-isoamyl | 62.3 | 1.65 | 0.66 |
| 4 |  |  | Saline (NaCl) | 71.1 | 1.93 | 2.33 |
|  |  |  | Wizard® Genomic - Promega | 13.8 | 1.3 | 0.43 |
|  |  |  | Phenol-chloroform-isoamyl | 33.6 | 1.44 | 0.66 |
| 5 |  |  | Saline (NaCl) | 137.9 | 1.21 | 0.87 |
|  |  |  | Wizard® Genomic - Promega | 8 | 1.04 | 0.29 |
|  |  |  | Phenol-chloroform-isoamyl | 27.1 | 1.76 | 0.32 |
| 1 | Tongue |  | Saline (NaCl) | 17.5 | 1.7 | 0.65 |
|  |  |  | Wizard® Genomic - Promega | 22.3 | 1.5 | 0.61 |
|  |  |  | Phenol-chloroform-isoamyl | 10.9 | 1.49 | 0.42 |
| 2 |  |  | Saline (NaCl) | 4.4 | 1.72 | 0.66 |
|  |  |  | Wizard® Genomic - Promega | 1.9 | 2.2 | -0.41 |
|  |  |  | Phenol-chloroform-isoamyl | 19.2 | 1.48 | 0.27 |
| 3 |  |  | Saline (NaCl) | 141.5 | 1.91 | 2.45 |
|  |  |  | Wizard® Genomic - Promega | 93.9 | 1.72 | 1.23 |
|  |  |  | Phenol-chloroform-isoamyl | 130.5 | 1.81 | 1.02 |
| 4 |  |  | Saline (NaCl) | 146.1 | 1.7 | 1.53 |
|  |  |  | Wizard® Genomic - Promega | 41 | 1.7 | 0.96 |
|  |  |  | Phenol-chloroform-isoamyl | 17.2 | 1.58 | 0.61 |
| 5 |  |  | Saline (NaCl) | 22.3 | 1.61 | 0.74 |
|  |  |  | Wizard® Genomic - Promega | 54.4 | 1.69 | 1.34 |
|  |  |  | Phenol-chloroform-isoamyl | 50.1 | 1.53 | 0.43 |
| 1 | Fin |  | Saline (NaCl) | 1.3 | 2.41 | 5.78 |
|  |  |  | Wizard® Genomic - Promega | 26 | 1.53 | 1.11 |
|  |  |  | Phenol-chloroform-isoamyl | 10.1 | 1.46 | 0.43 |
| 2 |  |  | Saline (NaCl) | 77.6 | 1.93 | 2.46 |
|  |  |  | Wizard® Genomic - Promega | 132.9 | 1.8 | 1.27 |
|  |  |  | Phenol-chloroform-isoamyl | 56.5 | 1.62 | 0.76 |
| 3 |  |  | Saline (NaCl) | 46.6 | 1.93 | 2.27 |
|  |  |  | Wizard® Genomic - Promega | 37.6 | 1.64 | 1.14 |
|  |  |  | Phenol-chloroform-isoamyl | 18.2 | 1.82 | 0.59 |
| 4 |  |  | Saline (NaCl) | 9.2 | 1.72 | 0.85 |
|  |  |  | Wizard® Genomic - Promega | 116.7 | 1.74 | 1.67 |
|  |  |  | Phenol-chloroform-isoamyl | 32.1 | 1.61 | 0.78 |
| 5 |  |  | Saline (NaCl) | 371.9 | 1.9 | 2.03 |
|  |  |  | Wizard® Genomic - Promega | 43.2 | 1.51 | 1.71 |
|  |  |  | Phenol-chloroform-isoamyl | 65.1 | 1.82 | 0.64 |
| 1 | Muscle | Alcohol | Saline (NaCl) | 16.8 | 2.04 | 1.53 |
|  |  |  | Wizard® Genomic - Promega | 33.3 | 1.57 | 0.84 |
|  |  |  | Phenol-chloroform-isoamyl | 17 | 1.68 | 0.43 |
| 2 |  |  | Saline (NaCl) | 171.3 | 1.84 | 2 |
|  |  |  | Wizard® Genomic - Promega | 20.3 | 1.42 | 0.32 |
|  |  |  | Phenol-chloroform-isoamyl | 18.6 | 1.51 | 0.29 |
| 3 |  |  | Saline (NaCl) | 3.7 | 1.04 | 0.26 |
|  |  |  | Wizard® Genomic - Promega | 1.1 | 0.91 | -0.04 |
|  |  |  | Phenol-chloroform-isoamyl | 37.3 | 1.59 | 0.5 |
| 4 |  |  | Saline (NaCl) | 60.8 | 1.9 | 2.18 |
|  |  |  | Wizard® Genomic - Promega | 28.8 | 1.59 | 0.84 |
|  |  |  | Phenol-chloroform-isoamyl | 20.3 | 1.64 | 0.46 |
| 5 |  |  | Saline (NaCl) | 38.9 | 1.9 | 1.81 |
|  |  |  | Wizard® Genomic - Promega | 12.6 | 1.17 | 0.35 |
|  |  |  | Phenol-chloroform-isoamyl | 50.5 | 1.53 | 0.5 |
| 1 | Tongue |  | Saline (NaCl) | 69.7 | 1.88 | 1.99 |
|  |  |  | Wizard® Genomic - Promega | 139 | 1.68 | 1.73 |
|  |  |  | Phenol-chloroform-isoamyl | 27.1 | 1.65 | 0.6 |
| 2 |  |  | Saline (NaCl) | 192.7 | 1.88 | 2.16 |
|  |  |  | Wizard® Genomic - Promega | 68.8 | 1.68 | 1.12 |
|  |  |  | Phenol-chloroform-isoamyl | 66.1 | 1.73 | 0.92 |
| 3 |  |  | Saline (NaCl) | 86 | 1.86 | 1.74 |
|  |  |  | Wizard® Genomic - Promega | 160.1 | 1.79 | 1.87 |
|  |  |  | Phenol-chloroform-isoamyl | 69.3 | 1.68 | 1.1 |
| 4 |  |  | Saline (NaCl) | 314.6 | 1.86 | 2.28 |
|  |  |  | Wizard® Genomic - Promega | 198.2 | 1.77 | 1.91 |
|  |  |  | Phenol-chloroform-isoamyl | 45.9 | 1.69 | 0.97 |
| 5 |  |  | Saline (NaCl) | 214.4 | 1.88 | 1.92 |
|  |  |  | Wizard® Genomic - Promega | 325.6 | 1.74 | 1.96 |
|  |  |  | Phenol-chloroform-isoamyl | 73.4 | 1.66 | 0.77 |
| 1 | Fin |  | Saline (NaCl) | 11.4 | 1.96 | 1.96 |
|  |  |  | Wizard® Genomic - Promega | 57.3 | 1.63 | 0.53 |
|  |  |  | Phenol-chloroform-isoamyl | 21.3 | 1.51 | 0.74 |
| 2 |  |  | Saline (NaCl) | 83.6 | 1.95 | 2.5 |
|  |  |  | Wizard® Genomic - Promega | 290 | 1.77 | 1.94 |
|  |  |  | Phenol-chloroform-isoamyl | 309.9 | 1.47 | 1.3 |
| 3 |  |  | Saline (NaCl) | 102.3 | 1.93 | 2.26 |
|  |  |  | Wizard® Genomic - Promega | 180.1 | 1.24 | 0.97 |
|  |  |  | Phenol-chloroform-isoamyl | 39.4 | 1.55 | 0.81 |
| 4 |  |  | Saline (NaCl) | 39.7 | 1.96 | 1.74 |
|  |  |  | Wizard® Genomic - Promega | 87.8 | 1.75 | 1.32 |
|  |  |  | Phenol-chloroform-isoamyl | 93.4 | 1.73 | 1.02 |
| 5 |  |  | Saline (NaCl) | 32.5 | 1.96 | 2.34 |
|  |  |  | Wizard® Genomic - Promega | 34.5 | 1.55 | 1.25 |
|  |  |  | Phenol-chloroform-isoamyl | 56.6 | 1.84 | 0.97 |
