# Supplementary material for: Quality analysis of genomic DNA and authentication of fisheries products based on distinct methods of DNA extraction
Source: PLoS One. 2023 Feb 28;18(2):e0282369. doi: 10.1371/journal.pone.0282369 (PMC9974130; doi:10.1371/journal.pone.0282369)
Supplement: S1 File — (DOCX) [file pone.0282369.s003.docx]

**File S1.** Summary of statistical analyses with mean, minimum, maximum and standard error (se) values of DNA quantification and purity ratios for each DNA extraction method in samples of *Lutjanus purpureus*.

**Saline (NaCl) method**

**DNA concentration (ng/µl)**

Data summary

| Tissue | Storage | Mean | Min | Max | se |
| --- | --- | --- | --- | --- | --- |
| Tongue | Alcohol | 176. | 69.7 | 315. | 44.9 |
| Fin | Frozen | 101. | 1.3 | 372. | 69.0 |
| Tongue | Frozen | 66.4 | 4.4 | 146. | 31.8 |
| Muscle | Alcohol | 58.3 | 3.7 | 171. | 31.8 |
| Muscle | Frozen | 55.1 | 2.6 | 138. | 29.9 |
| Fin | Alcohol | 53.9 | 11.4 | 102. | 16.9 |

Anova Two Way

|  | Df | Sum Square | Mean Square | F value | Pr(>F) |
| --- | --- | --- | --- | --- | --- |
| Storage | 1 | 3512 | 3512 | 0.442 | 0.513 |
| Tissue | 2 | 21463 | 10731 | 1.350 | 0.278 |
| Storage:Tissue | 2 | 31903 | 15952 | 2.007 | 0.156 |
| Residuals | 24 | 190734 | 7947 |  |  |

Post hoc Sidak

| Storage | Term | group1 | group2 | df | statistic | p | p.adj |
| --- | --- | --- | --- | --- | --- | --- | --- |
| Alcohol | Tissue | Fin | Muscle | 24 | -0.0780 | 0.938 | 1.00 |
| Alcohol | Tissue | Fin | Tongue | 24 | -2.16 | 0.0413 | 0.119 |
| Alcohol | Tissue | Muscle | Tongue | 24 | -2.08 | 0.0485 | 0.139 |
| Frozen | Tissue | Fin | Muscle | 24 | 0.820 | 0.420 | 0.805 |
| Frozen | Tissue | Fin | Tongue | 24 | 0.620 | 0.541 | 0.903 |
| Frozen | Tissue | Muscle | Tongue | 24 | -0.200 | 0.843 | 0.996 |

**A260/A280 ratio**

Data summary

| Tissue | Storage | Mean | Min | Max | se |
| --- | --- | --- | --- | --- | --- |
| Fin | Alcohol | 2 | 1.93 | 1.96 | 0.00583 |
| Fin | Frozen | 2 | 1.72 | 2.41 | 0.115 |
| Tongue | Alcohol | 1.9 | 1.86 | 1.88 | 0.00490 |
| Muscle | Alcohol | 1.7 | 1.04 | 2.04 | 0.179 |
| Muscle | Frozen | 1.7 | 1.21 | 1.97 | 0.146 |
| Tongue | Frozen | 1.7 | 1.61 | 1.91 | 0.0493 |

Anova Two Way

|  | Df | Sum Square | Mean Square | F value | Pr(>F) |
| --- | --- | --- | --- | --- | --- |
| Storage | 1 | 0.0116 | 0.01160 | 0.202 | 0.657 |
| Tissue | 2 | 0.2640 | 0.13200 | 2.294 | 0.123 |
| Storage:Tissue | 2 | 0.0419 | 0.02096 | 0.364 | 0.698 |
| Residuals | 24 | 1.3812 | 0.05755 |  |  |

Post hoc Sidak

| Storage | Term | group1 | group2 | df | statistic | p | p.adj |
| --- | --- | --- | --- | --- | --- | --- | --- |
| Alcohol | Tissue | Fin | Muscle | 24 | 1.37 | 0.183 | 0.455 |
| Alcohol | Tissue | Fin | Tongue | 24 | 0.527 | 0.603 | 0.937 |
| Alcohol | Tissue | Muscle | Tongue | 24 | -0.844 | 0.407 | 0.792 |
| Frozen | Tissue | Fin | Muscle | 24 | 1.54 | 0.136 | 0.355 |
| Frozen | Tissue | Fin | Tongue | 24 | 1.65 | 0.112 | 0.301 |
| Frozen | Tissue | Muscle | Tongue | 24 | 0.105 | 0.917 | 0.999 |

**A260/A230 ratio**

Data summary

| Tissue | Storage | Mean | Min | Max | se |
| --- | --- | --- | --- | --- | --- |
| Tongue | Alcohol | 1.7 | 1.12 | 1.96 | 0.154 |
| Fin | Frozen | 1.4 | 1.11 | 1.71 | 0.130 |
| Fin | Alcohol | 1.2 | 0.53 | 1.94 | 0.231 |
| Tongue | Frozen | 0.9 | 0.41 | 1.34 | 0.177 |
| Muscle | Alcohol | 0.5 | 0.04 | 0.84 | 0.157 |
| Muscle | Frozen | 0.5 | 0.29 | 0.73 | 0.0742 |

Anova Two Way

|  | Df | Sum Square | Mean Square | F value | Pr(>F) |
| --- | --- | --- | --- | --- | --- |
| Storage | 1 | 0.316 | 0.3162 | 2.438 | 0.1315 |
| Tissue | 2 | 4.458 | 2.2290 | 17.183 | 2.34e-05 |
| Storage:Tissue | 2 | 1.396 | 0.6978 | 5.379 | 0.0117 |
| Residuals | 24 | 3.113 | 0.1297 |  |  |

Post hoc Sidak

| Storage | Term | group1 | group2 | df | statistic | p | p.adj |
| --- | --- | --- | --- | --- | --- | --- | --- |
| Alcohol | Tissue | Fin | Muscle | 24 | 3.18 | 0.00405 | 0.0121 |
| Alcohol | Tissue | Fin | Tongue | 24 | -2.27 | 0.0328 | 0.0952 |
| Alcohol | Tissue | Muscle | Tongue | 24 | -5.44 | 0.0000136 | 0.0000407 |
| Frozen | Tissue | Fin | Muscle | 24 | 3.90 | 0.000681 | 0.00204 |
| Frozen | Tissue | Fin | Tongue | 24 | 2.06 | 0.0501 | 0.143 |
| Frozen | Tissue | Muscle | Tongue | 24 | -1.84 | 0.0789 | 0.219 |

**Wizard® Genomic - Promega method**

**DNA concentration (ng/µl)**

Data summary

| Tissue | Storage | Mean | Min | Max | se |
| --- | --- | --- | --- | --- | --- |
| Tongue | Alcohol | 178. | 68.8 | 326. | 42.4 |
| Fin | Alcohol | 130. | 34.5 | 290 | 47.1 |
| Fin | Frozen | 71.3 | 26 | 133. | 22.2 |
| Tongue | Frozen | 42.7 | 1.9 | 93.9 | 15.6 |
| Muscle | Frozen | 24 | 8 | 39.3 | 5.97 |
| Muscle | Alcohol | 19.2 | 1.1 | 33.3 | 5.76 |

Anova Two Way

|  | Df | Sum Square | Mean Square | F value | Pr(>F) |
| --- | --- | --- | --- | --- | --- |
| Storage | 1 | 29944 | 29944 | 7.462 | 0.01163 |
| Tissue | 2 | 47503 | 23752 | 5.919 | 0.00814 |
| Storage:Tissue | 2 | 24710 | 12355 | 3.079 | 0.06453 |
| Residuals | 24 | 96307 | 4013 |  |  |

Post hoc Sidak

| Storage | Term | group1 | group2 | df | statistic | p | p.adj |
| --- | --- | --- | --- | --- | --- | --- | --- |
| Alcohol | Tissue | Fin | Muscle | 24 | 2.76 | 0.0108 | 0.0321 |
| Alcohol | Tissue | Fin | Tongue | 24 | -1.21 | 0.239 | 0.559 |
| Alcohol | Tissue | Muscle | Tongue | 24 | -3.97 | 0.000566 | 0.00170 |
| Frozen | Tissue | Fin | Muscle | 24 | 1.18 | 0.249 | 0.577 |
| Frozen | Tissue | Fin | Tongue | 24 | 0.713 | 0.483 | 0.861 |
| Frozen | Tissue | Muscle | Tongue | 24 | -0.468 | 0.644 | 0.855 |

**A260/A280 ratio**

Data summary

| Tissue | Storage | Mean | Min | Max | se |
| --- | --- | --- | --- | --- | --- |
| Tongue | Frozen | 1.8 | 1.5 | 2.2 | 0.116 |
| Tongue | Alcohol | 1.7 | 1.68 | 1.79 | 0.0227 |
| Fin | Alcohol | 1.6 | 1.24 | 1.77 | 0.0958 |
| Fin | Frozen | 1.6 | 1.51 | 1.8 | 0.0568 |
| Muscle | Frozen | 1.4 | 1.04 | 1.65 | 0.105 |
| Muscle | Alcohol | 1.3 | 0.91 | 1.59 | 0.129 |

Anova Two Way

|  | Df | Sum Square | Mean Square | F value | Pr(>F) |
| --- | --- | --- | --- | --- | --- |
| Storage | 1 | 0.0183 | 0.0183 | 0.404 | 0.53097 |
| Tissue | 2 | 0.7621 | 0.3810 | 8.437 | 0.00168 |
| Storage:Tissue | 2 | 0.0014 | 0.0007 | 0.016 | 0.98412 |
| Residuals | 24 | 1.0840 | 0.0452 |  |  |

Post hoc Sidak

| Storage | Term | group1 | group2 | df | statistic | p | p.adj |
| --- | --- | --- | --- | --- | --- | --- | --- |
| Alcohol | Tissue | Fin | Muscle | 24 | 1.90 | 0.0689 | 0.193 |
| Alcohol | Tissue | Fin | Tongue | 24 | -1.07 | 0.295 | 0.649 |
| Alcohol | Tissue | Muscle | Tongue | 24 | -2.98 | 0.00657 | 0.0196 |
| Frozen | Tissue | Fin | Muscle | 24 | 1.86 | 0.0752 | 0.209 |
| Frozen | Tissue | Fin | Tongue | 24 | -0.878 | 0.389 | 0.772 |
| Frozen | Tissue | Muscle | Tongue | 24 | -2.74 | 0.0115 | 0.0340 |

**A260/A230 ratio**

Data summary

| Tissue | Storage | Mean | Min | Max | se |
| --- | --- | --- | --- | --- | --- |
| Tongue | Alcohol | 1.7 | 1.12 | 1.96 | 0.154 |
| Fin | Frozen | 1.4 | 1.11 | 1.71 | 0.130 |
| Fin | Alcohol | 1.2 | 0.53 | 1.94 | 0.231 |
| Tongue | Frozen | 0.9 | 0.41 | 1.34 | 0.177 |
| Muscle | Alcohol | 0.5 | 0.04 | 0.84 | 0.157 |
| Muscle | Frozen | 0.5 | 0.29 | 0.73 | 0.0742 |

Anova Two Way

|  | Df | Sum Square | Mean Square | F value | Pr(>F) |
| --- | --- | --- | --- | --- | --- |
| Storage | 1 | 0.316 | 0.3162 | 2.438 | 0.1315 |
| Tissue | 2 | 4.458 | 2.2290 | 17.183 | 2.34e-05 |
| Storage:Tissue | 2 | 1.396 | 0.6978 | 5.379 | 0.0117 |
| Residuals | 24 | 3.113 | 0.1297 |  |  |

Post hoc Sidak

| Storage | Term | group1 | group2 | df | statistic | p | p.adj |
| --- | --- | --- | --- | --- | --- | --- | --- |
| Alcohol | Tissue | Fin | Muscle | 24 | 3.18 | 0.00405 | 0.0121 |
| Alcohol | Tissue | Fin | Tongue | 24 | -2.27 | 0.0328 | 0.0952 |
| Alcohol | Tissue | Muscle | Tongue | 24 | -5.44 | 0.0000136 | 0.0000407 |
| Frozen | Tissue | Fin | Muscle | 24 | 3.90 | 0.000681 | 0.00204 |
| Frozen | Tissue | Fin | Tongue | 24 | 2.06 | 0.0501 | 0.143 |
| Frozen | Tissue | Muscle | Tongue | 24 | -1.84 | 0.0789 | 0.219 |

**Phenol-chloroform-isoamyl method**

**DNA concentration (ng/µl)**

Data summary

| Tissue | Storage | Mean | Min | Max | se |
| --- | --- | --- | --- | --- | --- |
| Fin | Alcohol | 104. | 21.3 | 310. | 52.8 |
| Tongue | Alcohol | 56.4 | 27.1 | 73.4 | 8.71 |
| Tongue | Frozen | 45.6 | 10.9 | 130. | 22.3 |
| Fin | Frozen | 36.4 | 10.1 | 65.1 | 10.7 |
| Muscle | Frozen | 34.6 | 18.3 | 62.3 | 7.41 |
| Muscle | Alcohol | 28.7 | 17 | 50.5 | 6.55 |

Anova Two Way

|  | Df | Sum Square | Mean Square | F value | Pr(>F) |
| --- | --- | --- | --- | --- | --- |
| Storage | 1 | 4397 | 4397 | 1.477 | 0.236 |
| Tissue | 2 | 7446 | 3723 | 1.251 | 0.304 |
| Storage:Tissue | 2 | 7444 | 3722 | 1.250 | 0.304 |
| Residuals | 24 | 71447 | 2977 |  |  |

Post hoc Sidak

| Storage | Term | group1 | group2 | df | statistic | p | p.adj |
| --- | --- | --- | --- | --- | --- | --- | --- |
| Alcohol | Tissue | Fin | Muscle | 24 | 2.18 | 0.0389 | 0.112 |
| Alcohol | Tissue | Fin | Tongue | 24 | 1.38 | 0.179 | 0.447 |
| Alcohol | Tissue | Muscle | Tongue | 24 | -0.800 | 0.431 | 0.816 |
| Frozen | Tissue | Fin | Muscle | 24 | 0.0522 | 0.959 | 1.00 |
| Frozen | Tissue | Fin | Tongue | 24 | -0.266 | 0.792 | 0.991 |
| Frozen | Tissue | Muscle | Tongue | 24 | -0.318 | 0.753 | 0.985 |

**A260/A280 ratio**

Data summary

| Tissue | Storage | Mean | Min | Max | se |
| --- | --- | --- | --- | --- | --- |
| Fin | Frozen | 1.7 | 1.46 | 1.82 | 0.0690 |
| Tongue | Alcohol | 1.7 | 1.65 | 1.73 | 0.0139 |
| Fin | Alcohol | 1.6 | 1.47 | 1.84 | 0.0707 |
| Muscle | Alcohol | 1.6 | 1.51 | 1.68 | 0.0321 |
| Muscle | Frozen | 1.6 | 1.4 | 1.76 | 0.0665 |
| Tongue | Frozen | 1.6 | 1.48 | 1.81 | 0.0606 |

Anova Two Way

|  | Df | Sum Square | Mean Square | F value | Pr(>F) |
| --- | --- | --- | --- | --- | --- |
| Storage | 1 | 0.0067 | 0.00675 | 0.425 | 0.521 |
| Tissue | 2 | 0.0269 | 0.01344 | 0.846 | 0.442 |
| Storage:Tissue | 2 | 0.0281 | 0.01407 | 0.885 | 0.426 |
| Residuals | 24 | 0.3816 | 0.01590 |  |  |

Post hoc Sidak

| Storage | Term | group1 | group2 | df | statistic | p | p.adj |
| --- | --- | --- | --- | --- | --- | --- | --- |
| Alcohol | Tissue | Fin | Muscle | 24 | 0.376 | 0.710 | 0.976 |
| Alcohol | Tissue | Fin | Tongue | 24 | -0.777 | 0.444 | 0.829 |
| Alcohol | Tissue | Muscle | Tongue | 24 | -1.15 | 0.260 | 0.595 |
| Frozen | Tissue | Fin | Muscle | 24 | 1.35 | 0.188 | 0.465 |
| Frozen | Tissue | Fin | Tongue | 24 | 1.10 | 0.281 | 0.628 |
| Frozen | Tissue | Muscle | Tongue | 24 | -0.251 | 0.804 | 0.992 |

**A260/A230 ratio**

Data summary

| Tissue | Storage | Mean | Min | Max | se |
| --- | --- | --- | --- | --- | --- |
| Fin | Alcohol | 1 | 0.74 | 1.3 | 0.0974 |
| Tongue | Alcohol | 0.9 | 0.6 | 1.1 | 0.0861 |
| Fin | Frozen | 0.6 | 0.43 | 0.78 | 0.0635 |
| Tongue | Frozen | 0.6 | 0.27 | 1.02 | 0.129 |
| Muscle | Frozen | 0.5 | 0.32 | 0.66 | 0.0623 |
| Muscle | Alcohol | 0.4 | 0.29 | 0.5 | 0.0388 |

Anova Two Way

|  | Df | Sum Square | Mean Square | F value | Pr(>F) |
| --- | --- | --- | --- | --- | --- |
| Storage | 1 | 0.2503 | 0.25025 | 6.978 | 0.01429 |
| Tissue | 2 | 0.5310 | 0.26552 | 7.403 | 0.00313 |
| Storage:Tissue | 2 | 0.3039 | 0.15196 | 4.237 | 0.02655 |
| Residuals | 24 | 0.8608 | 0.03586 |  |  |

Post hoc Sidak

| Storage | Term | group1 | group2 | df | statistic | p | p.adj |
| --- | --- | --- | --- | --- | --- | --- | --- |
| Alcohol | Tissue | Fin | Muscle | 24 | 4.44 | 0.000172 | 0.000515 |
| Alcohol | Tissue | Fin | Tongue | 24 | 0.802 | 0.431 | 0.815 |
| Alcohol | Tissue | Muscle | Tongue | 24 | -3.64 | 0.00130 | 0.00390 |
| Frozen | Tissue | Fin | Muscle | 24 | 0.852 | 0.403 | 0.787 |
| Frozen | Tissue | Fin | Tongue | 24 | 0.751 | 0.460 | 0.842 |
| Frozen | Tissue | Muscle | Tongue | 24 | -0.100 | 0.921 | 1.00 |
